# Treating childhood cancer in Rwanda: the nephroblastoma example

**DOI:** 10.11604/pamj.2015.21.326.5912

**Published:** 2015-08-31

**Authors:** Aimable Kanyamuhunga, Lisine Tuyisenge, Daniela Cristina Stefan

**Affiliations:** 1 Department of Pediatrics and Child Health, Kigali University Teaching Hospital, Rwanda; 2 Department of Pediatrics and Child Health, Tygerberg Hospital, Stellenbosch University, Cape Town, South Africa; 3 South African Medical Research Council, Parrow, Cape Town, South Africa

**Keywords:** Cost, nephroblastoma, Rwanda, childhood cancer, Africa

## Abstract

**Introduction:**

Wilms tumor (WT) or nephroblastoma is the commonest childhood cancer in Rwanda. Nephroblastoma is regarded as one of the successes of pediatric oncology with long-term survival approaching 90%. The Objectives to evaluate the feasibilityof treating childhood cancer using the nephroblastoma example and to calculate its cost of treatment in Rwanda.

**Methods:**

Prospective study over a 2 year period: 01 Jan 2010- 31 December 2011. A questionnaire was completed by all participants in the study and the following variables were collected at Kigali University Teaching Hospital: age at diagnosis, gender, transport cost, cost of investigations, staging, treatment and outcome, cost of hospitalization, type of medical, surgical, radiological interventions and their costs, number of admissions per patient and factors related to non compliance to treatment. All patients had a confirmed diagnosis on histopathology examination. The cost for treatment was calculated for early and late stage and was expressed in USA dollars. Analysis was done with SPSS 16.0.

**Results:**

There were 25 patients diagnosed and treated for WT during the study period. Almost half of the patients 14/25 (56%) had advanced disease, seven children (28%) had stage IV, seven children stage III, six patients (24%) with stage II, while the remaining five (20%) had stage I with high risk tumor. The direct cost of management ranged from1,831.2 USD for early disease to 2,418.7 USD for advanced disease. The cost of transport, investigations and drugs were recorded as main contributing factors to the feasibility and cost of the treatment in 80% of the responses, followed by late presentation (56%) and poor compliance to treatment.

**Conclusion:**

Most challenges are related to unaffordable treatment and late presentation. The management of WT is feasible in Rwandan setting but efforts should be made in order to improve awareness of childhood cancer, early diagnosis and access to care. The government of Rwanda is committed to improve cancer care in the country and organized the first pediatric international oncology conference in Kigali, in March 2012 to develop National protocols for the top five most common cancers in children.

## Introduction

The nephroblastoma or Wilms tumor (WT)is the commonest solid childhood renal tumor, accounting for about 6% of all pediatric malignancies [1]. It predominantly affects children less than five years of age, with 90% of new cases diagnosed before age of three years [1, 2]. The cancer treatment in childhood is expensive especially in the Low-income countries with a low and very low GDP per capita. Cost of illness includes three categories: Direct, Indirect, and Psychosocial costs [3]. Typically it involves periods of hospitalization,time spent by physician and other professional services, use of laboratory and other diagnostic tests, administration of chemotherapy, use of other pharmaceuticals and surgical or radiotherapy approach.

All these components impose a large financial burden on health-care systems and on the families of the children who are affected [4]. Rwanda is a Low-income country, with a GDP per capita which averaged 782.2 USD from 1980 until 2012 and reached an all time high of 1167.2 USD in December 2012 [5]. Rwanda made progress in reducing infection-related childhood deaths over the last two decades. More efforts are now directed towards addressing the burden of non-communicable diseases and cancer represents one of them. Childhood cancer is not a common disease but is curable if diagnosed early and treated appropriately. In this context it is essential to estimate the current cancer burden and the feasibility of treatment of childhood cancer particularly for the most frequent and curable types. This study on Wilms tumor at Kigali University Teaching Hospital is the first cost and feasibility study on childhood cancer in Rwanda.

### Objectives

The objectives of the study were the calculation of the direct cost of treatment of Wilms tumor in Rwanda, the evaluation of feasibility of treatment and identification of the major obstacles of treating this disease.

## Methods

It was a hospital based prospective descriptive study of patients with nephroblastoma treated in the pediatric department of Kigali University Teaching Hospital within 2 years period from the 01 January 2010-31 December 2011. The KUTH is located in the Center of Kigali city and is the main public health institution in Rwanda. During the study period the hospital was the only public referral hospital in the country where childhood cancer was diagnosed and treated. It serves normally one to two million people from a largely urban and rural area, children representing more than 40% of the population.

The patients were followed during the period of this study, and readmitted several times in the Pediatric Oncology Unit (POU)according to their chemotherapy schedule or if there were other medical indications (infections, anemia or thrombocytopenia which needed blood or blood products transfusions, neutropenia, etc). The ward has a capacity of 12 beds dedicated to oncology and the occupation rate is almost always close to 100%. The unit admits an average of 30-40newly diagnosed cancer patients per year.

### Eligibility criteria

All patients admitted and diagnosed with Wilms tumor at Kigali University Teaching Hospital from 01 January 2010 - 31 December 2011were included in the study. Parents or legal guardians of children enrolled in this study signed a written consent form. Patients, who died before the confirmation of the diagnosis, were excluded from the study.

### Protocol used

After a thorough physical examination, the routine biochemical panel (renal function tests, liver function tests), viral panel included (Hepatitis A, B, C and HIV), full blood count, urinalysis completed by the following imaging: chest radiography, abdominal ultrasound, heart ultrasonography and abdominal computer tomography (CT) scan were done for all patients enrolled in this study as baseline assessment. The patients were started on preoperative chemotherapy by using the SIOP 2001 Nephroblastoma protocol (Table 1). For surgical purpose, abdominal ultrasound was repeated for all cases after the preoperative chemotherapy course.

**Table 1:** Preoperative chemotherapy SIOP 2001

AMD	45ug/kg	IVI push	↓		↓		↓	
VCR	1.5mg/m^2^	IVI push	↓	↓	↓	↓	↓	↓
DOX	50mg/m^2^		↓				↓	
								
		WEEKS	1	2	3	4	5	6

AMD – Actinomycin; VCR – Vincristine; DOX – Doxorubicin

In the following one to two weeks after preoperative chemotherapy, the patient was operated. If the time of surgery exceeded 3 weeks after finishing the administration of chemotherapy and the patient had no other contraindications to postpone the nephrectomy, the delay was considered surgical delay. If the time for final pathology report exceeded 3 weeks the delay was considered delay in histopathology. The patient was readmitted in the POU after surgery and started on a postoperative chemotherapy depending on the staging report.

At the beginning of treatment, opportunistic infections constituted the main reason of admission of the patients but once the chemotherapy started the side effects predominated the clinical picture of admissions. The routinely tests before administration of chemotherapy cycle were: full blood count (FBC), renal function tests and electrolytes. Seven to ten days after chemotherapy administration, patients had blood counts taken either at the nearest health facility or at KUTH.

### Cost calculation

The cost was calculated using 2010-2011 tariffs applied by the hospital. The cost items have been identified and activity reports were used for calculating the total costs using unit prices and occurrences. We consulted the Accounting Department of UTH-Kigali in order to get the price of every variable concerned. It consisted of costs of hospitalization, chemotherapy drugs and supportive treatment like transfusions and intravenous fluid used, investigations, surgical intervention and transport fees. However the transport fees were not included into calculations in determining the total cost as it differed from patient to patient, but counted as a contributing factor to the treatment adherence. The cost for transport was calculated separately and we consulted the price of common bus transport regulation agency available in 2011.

The total cost presented in this paper did not take in consideration the time spent by the medical staff with each patient or their salaries. The currency used was in Rwandan francs and the costs were converted in United States Dollars (USD) at the exchange rate that was on market at the end of December 2011(1 USD= 605 RFW).

### Factors related to treatment compliance

The data were collected on a structured standardized questionnaire based on the following variables recorded in the specific cancer file for each patient: cost of drugs and affordability of chemotherapy, antibiotics and supportive care, date of surgical intervention in relationship to the end of chemotherapy protocol, date of release of the pathology report, withdrawal from chemotherapy, disease stage at enrollment and post surgery, number of visits for investigations and indicated admissions. Other data that were not available in patient's records were obtained through interviews with mother or next kin of the patient by the Principal investigator (lack of transport fees, illness before date of appointment, etc.)

### Ethical approval

The data collected in this study did not include any personal identifiers as they were stored and protected with a password. The data access was limited to the Principal investigator and was anonymously analyzed. The project proposal was presented to Research committee of the Faculty of Medicine and it was also submitted to UTH-Kigali's Ethical and Research committees. The approval letters have been obtained from both Research and Ethical committees. The parents or guardians of the patients signed a consent form for participation in this study.

## Results

There were 25 patients (15 males and 10 females, sex ratio 1,5:1) diagnosed and treated for Wilms tumor in Pediatric Oncology Unit of Kigali University Teaching Hospital during the study period. The average age of our patients at presentation was 4,7 years (2-12 years at diagnosis). The one patient who was 12 years old had the histology revised and confirmed by a team of pathologists. Almost half of the patients 14/25 representing 56% presented with advanced disease (stage 3 and 4) and 28% of them presented with metastases.

The patients with the Community Health Insurance ‘‘ Mutuelle de Santé ” needed to pay 10% of the total cost (the transport fee is not covered by the Mutuelle) therefore was not taken into consideration in calculating the total cost. The cost for management ranged from 1,831.2 US$ for early stage to 2,418.7 US$ for advanced disease stage (radiotherapy was not included as it is not available in Rwanda). The cost of medications representing 38.7%, laboratories 25.7% followed by in-hospital stay 16.6% were the most important drivers of overall costs, and were higher in the patients presenting with advanced disease compared to early presentation. The cost difference between early and late presentation were mainly attributed to the more intensive chemotherapy required for advanced stage disease thus longer in-hospital stay, the more episodes of sepsis, anemia and thrombocytopenia needing more admissions and therapy, as well as the laboratories work-up in due of chemotherapy side effects monitoring.

The management of patients with Nephroblastoma at Kigali University Teaching Hospital was affected by several factors such as unaffordable treatment due to poverty representing 80% followed by poor compliance to treatment due to lack of transport fees in 72% and late presentation in 56%. More than half of the mothers of the patients (18 out 25) described the lack of financial funds as the main reason for not affording the transport (Table 2). In 6 cases the histopathology report was released with a delay of more than 3 weeks after the operation and 2 patients with advanced diseases were operated later than 2 weeks from the last preoperative chemotherapy due to co morbidities and severe malnutrition.

**Table 2:** Characteristics of major obstacles of treating successfully the WT in our settings

Characteristics	Number	%
Lack of money for transport	18/25	72
Lack of money for medication	20/25	80
Delayed surgical intervention	2/25	8
Delayed histopathology exam's report	6/25	24
Withdraw from chemotherapy	5/25	20
Patients consult with advanced disease	14/25	56

**Figure 1:**
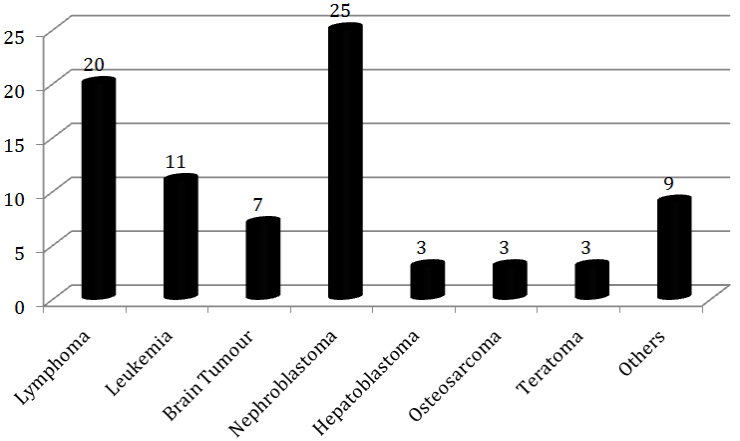
Wilms tumor and other malignancies seen in POU of the UTH-Kigali

During the period of 2 years, 81 children have been diagnosed with malignancies in the Paediatric Department of Kigali University Teaching Hospital. The most common pathology was Nephroblastoma with 25 cases, followed by Lymphoma with 20 cases and Leukaemia with 11 cases. Abdominal distension was the most common clinical presentation (Figure 1).

## Discussion

The Wilms tumor or Nephroblastoma is the commonest childhood cancer in Rwanda. The peak age incidence of 2-5 years and the sex ratio in this study is similar to other international studies [6]. The distribution of the stages in Rwanda reflects the general situation of most patients in numerous African countries that is late and with advanced disease. Late presentation is a common problem in developing countries and may be probably related mostly to ignorance and poverty [7].

In addition to that, most of these children are usually first managed in a primary care center where facilities and qualified personnel needed for early diagnosis are missing. The Kigali University Teaching Hospital, in the period under review, was the only one referral public Hospital taking care of these children with cancer in the country therefore we received patients from all over the country thus transport was a vital issue adding to the cost as well as the delay in diagnosis; this was similar to the results reported elsewhere [7]. Collaborative efforts among Surgeons, Pathologists, Pediatricians, and Radiologists have been regarded as a major factor in excellent outcome from current management of WT [6, 8]. The multidisciplinary approach was impaired in the management of WT in the period under review; the delay in surgical intervention and histopathology report was 8% and 24% respectively. The definitive management of WT involves surgery, chemotherapy and radiotherapy for the patients with advanced stage disease. In our study, none of our patients had radiotherapy, as radiotherapy machines are not available in the country. The nephrectomy was performed by a general surgeon. Our study highlighted the difference in cost of managing a patient with advanced disease versus early presentation. The cost difference was mainly attributed to the more intensive chemotherapy required for advanced stage disease thus longer in-hospital stay as well as the laboratories work-up in due of chemotherapy side effects monitoring. For Rwanda a country with an average GDP per capita of 782.2 USD until 2012the management of nephroblastoma remains very expensive in our settings.

The cost was mostly driven by the high cost of chemotherapy drugs and the lack of money for transport for the patients. A poor compliance of the treatment, following the chemotherapy regimen was described in similar studies in Sudan [7]. Our findings differ from previous studies reported in the literature due to the fact that pathology, radiotherapy, chemotherapy drugs, and in-hospital days in Rwanda are not free services or at a very minimal cost [9].

Nephroblastoma is regarded as one of the successes of pediatric oncology with long-term survival approaching 90% and 70% for localized and metastatic diseases [6, 10]. Out of 25 patients treated for Wilms tumor at Kigali University Teaching Hospital, five patients (20%) withdrew with the remaining completing the chemotherapy protocol. More than half of them presented with advanced disease. The overall survival is not known as many patients were lost to follow up. The rate of abandonment in our settings is a reflection of problems peculiar to the least developed and developing countries, problems with fundamental issues such as poverty, ignorance, and high cost of the drugs with inadequate and inefficient supply.

## Conclusion

The results from our study present the real costs of the most common childhood cancer in Rwanda and call for awareness and early diagnosis. Factors such as advance disease, poor compliance to treatment, poverty, expensive chemotherapy, lack of trained staff and multidisciplinary collaboration constitute major challenges in managing our cancer patients. In this context the Rwandan government organized an international conference in Kigali, in 2012 with the theme: “ACTING TODAY FOR HEALTH TOMORROW”. One of the aims was to adopt national guidelines for the treatment of major childhood cancers. The management of Nephroblastoma is feasible in our setting with multidisciplinary collaboration but special efforts should be done to increase awareness, early diagnosis and to reduce the rate of abandonment. Free health care for children with cancer and establishment of collaboration with a tertiary cancer Center in the developed World in a form of twinning are some of the options to improve survival of children with cancer in African countries.
